# Ravulizumab use for acetylcholine receptor-positive generalized myasthenia gravis in clinical practice

**DOI:** 10.3389/fneur.2024.1378080

**Published:** 2024-06-11

**Authors:** Nakul Katyal, Raghav Govindarajan, Neelam Goyal, Suraj Muley, Srikanth Muppidi

**Affiliations:** ^1^Department of Neurology, University of Kentucky, Lexington, KY, United States; ^2^Department of Neurology, HSHS Medical Group, O'Fallon, IL, United States; ^3^Department of Neurology, Stanford Healthcare, Stanford, CA, United States; ^4^Department of Neurology, Honor Health, Bob Bove Neurosciences Institute, Scottsdale, AZ, United States

**Keywords:** acetylcholine receptor antibody positive, generalized myasthenia gravis, ravulizumab, MG-ADL, complement inhibition

## Abstract

**Purpose:**

To describe the early experience of ravulizumab use in acetylcholine receptor antibody-positive generalized myasthenia gravis (AChR+ve gMG).

**Methods:**

This multicenter retrospective study included AChR+ve gMG patients who were treated with ravulizumab and had both pre- and post-ravulizumab myasthenia gravis activities of daily living (MG-ADL) scores. Clinical information regarding MG history, concomitant treatment(s), MG-ADL, other MG-specific measures, and adverse events were recorded.

**Results:**

A total of 18 patients with mean age of 61.83 (±16.08, *n* = 18) years were included in this cohort. In 10 complement inhibitor naive patients, a clinically meaningful reduction in mean Mg-ADL (baseline: 6.6 (±3.58) vs. 4.4 (±2.28), post ravulizumab) was seen. 6 out of 10 patients (60%) had clinically meaningful reduction post ravulizumab and two achieved minimum symptom expression (MSE). In 8 patients switched from eculizumab to ravulizumab, further reduction was noted in post ravulizumab mean MG-ADL (Baseline: 3.25 (±3.34) vs. 1.5 (±2.34) post ravulizumab). None of the patients who switched from eculizumab to ravulizumab experienced worsening symptoms. Eleven out of 14 (78.5%) patients on prednisone therapy were able to reduce their prednisone dose post-ravulizumab. None of the patients experienced any major side effects.

**Conclusion:**

In our clinical practice, 60% of AChR+ve gMG complement inhibitor naive patients experienced a clinically meaningful improvement in MG-ADL scores with ravulizumab. Patients were safely switched from eculizumab to ravulizumab and had further improvement in their mean MG-ADL scores. Of those on prednisone therapy, the majority were able to reduce their prednisone dosage.

## 1 Introduction

The past 5 years have seen a significant increase in the number of FDA-approved therapies for the treatment of myasthenia gravis (MG). Since 2017, three complement inhibitors and two neonatal FC receptor (FcRn) antagonists have been approved for management of MG (1, 2). The first complement inhibitor to receive FDA approval for MG was eculizumab, a monoclonal antibody targeting C5 (3). In April 2022, a longer-acting form of C5 monoclonal antibody, ravulizumab was approved by the FDA for acetylcholine receptor antibody-positive generalized myasthenia gravis (AChR+ve gMG) patients (4). Although ravulizumab and eculizumab have similar mechanisms of action, ravulizumab has a longer half-life (51 vs. 14 days) and consequently has a lower infusion burden (5). Even though the mechanism of action of these two complement inhibitors is similar, clinical outcomes in the trial have subtle differences. The patient population in the eculizumab clinical trial had met the criteria for refractory AChR+ve MG, but this was not a requirement for the ravulizumab clinical trial. Additionally, unlike clinical trials for other hematological conditions such as paroxysmal nocturnal hemoglobinuria, patients with prior complement therapy were not included in the ravulizumab clinical trial (4–6). Therefore, the clinical effectiveness of switching from eculizumab to ravulizumab, as well as the effectiveness of ravulizumab in a diverse MG patient population is unclear even though recent publication highlights benefit in the long-term follow-up of phase 3 clinical trial patient population (7). To shed light on these questions, we report our clinical experience of ravulizumab from three large neuromuscular practices.

## 2 Materials and methods

### 2.1 Study design

Patients were identified through the neuromuscular practices of the investigators. Inclusion criteria were AChR+ve gMG, age ≥ 18 years, received at least one dose of ravulizumab, and had MG-ADL scores before and after ravulizumab treatment. We obtained patient information regarding MG-specific history, antibody status, history of thymoma, and thymectomy. MG-specific therapy at the time of ravulizumab initiation was obtained with special attention to any patients switching from eculizumab to ravulizumab or efgartigimod to ravulizumab. Patients who were not on complement inhibitor therapy prior to initiation of ravulizumab were considered complement inhibitor naive. Patients who were started on ravulizumab from May 2022 to May 2023 were included in this analysis.

### 2.2 Outcome measures

The main outcome measure for clinical effectiveness used in this analysis was MG-ADL, a primary outcome measure in most clinical trials (8). We assessed both clinically meaningful improvement in ADL and also a number of patients who achieved Minimum Symptom Expression (MSE, a MG-ADL score of 0 or 1). We also documented the changes in prednisone dose or other immunosuppressive therapies after starting ravulizumab. We reviewed any reported adverse events during the clinical evaluation. This study was approved by the Institutional Review Board at each institution.

## 3 Results

### 3.1 Baseline characteristics

A total of 18 patients with a men age of 61.83 (±16.08, *n* = 18) years were included in this cohort. Among the 18, 11 were male and 7 were female. 16 patients were Caucasian and the remaining 2 were Hispanic.

10 out of 18 patients were complement inhibitor naive and were on corticosteroids and or corticosteroid sparing immunosuppressants prior to initiation of ravulizumab. Eight patients were transitioned from eculizumab to ravulizumab. The mean interval from MG diagnosis to ravulizumab initiation was 6.30 (±3.74, *n* = 13) years.

Seven patients had baseline cardiac comorbidities including hypertension, hyperlipidemia, atrial fibrillation, atrial flutter, and right bundle branch block. Four patients had diabetes, two patients had underlying pulmonary disease including chronic obstructive pulmonary disease (COPD) and obstructive sleep apnea (OSA) and one patient had breast cancer. Two patients with thymoma had undergone thymectomy prior to ravulizumab use. Three other patients without thymoma had prior thymectomies. The mean (SD) duration between thymectomy and initiation of ravulizumab was 4 (± 3.28; *n* = 5) years.

Prior to ravulizumab use, 14 patients were on prednisone with a mean dose of 19.21 (±15.72) mg/day. Nine patients were on steroid-sparing agents (5 on mycophenolate and 4 on azathioprine) and 3 patients were on maintenance intravenous immunoglobulins (IVIG). Additionally, 8 patients were on eculizumab and 3 were on efgartigimod at baseline and were then switched to ravulizumab. Baseline characteristics are shown in Table 1.

**Table 1:** Baseline demographics and MG treatments in 18 patients in the cohort.

Age	61.83 (±16.08, *n* = 18) Y
Gender	11 M, 7 F
Thymoma	2
Thymectomy	5 (2 with thymoma, 3 non thymoma)
Disease duration	6.30 (±3.74, *n* = 13) Y
MG Immunotherapy at time of ravulizumab initiation	*N* (%)
Prednisone	14 (77.7%)
Mycophenolate	5 (27.7%)
Azathioprine	4 (22.2%)
Eculizumab	8 (44.4%)
Efgartigimod	3 (16.6%)
Maintenance IVIG	3 (16.6%)
Clinical Scores	Mean (SD, *n*)
Mean MG-ADL	5.05 (±3.84, *n* = 18)

### 3.2 Clinical outcomes

#### 3.2.1 MG-ADL score

##### 3.2.1.1 Treatment response in complement inhibitor naive patients

In 10 complement inhibitor naive patients, a clinically meaningful reduction in mean Mg-ADL (baseline: 6.4 (±3.74) vs. 4.4 (±2.4), post ravulizumab) was seen. 6 out of 10 (60%) patients noted a clinically meaningful reduction in MG-ADL score (>2 point change) and two (20%) patients achieved MSE. The mean time from loading dose to post ravulizumab MG-ADL assessment was 4.6 (±1.26) months. Disease characteristics pre and post ravulizumab for complement inhibitor naive patients (baseline MG-ADL >1) are shown in Table 2.

**Table 2:** Disease characteristics pre and post ravulizumab for complement inhibitor naive patients.

Parameters	Mean MG-ADL at baseline	Post ravulizumab Mean MG-ADL (SD)	Mean duration from loading dose to assessment (months)	Minimum symptom expression (MSE) (N)	Clinically meaningful reduction >2 MG-ADL (N)
All patients *n* = 10	6.4 (±3.74)	4.4 (±2.4)	4.6 (±1.26)	2	6
Patients previously on efgartigimod *n* = 3	7.66 (±3.7)	5.33 (±1.24)	4.33 (±0.47)	0	1
Patients previously not on efgartigimod *n* = 7	5.85 (±3.57)	4 (±2.7)	4.42 (±0.97)	2	5

###### 3.2.1.1.1 Patients switched from efgartigimod to ravulizumab

**Figure 1:**
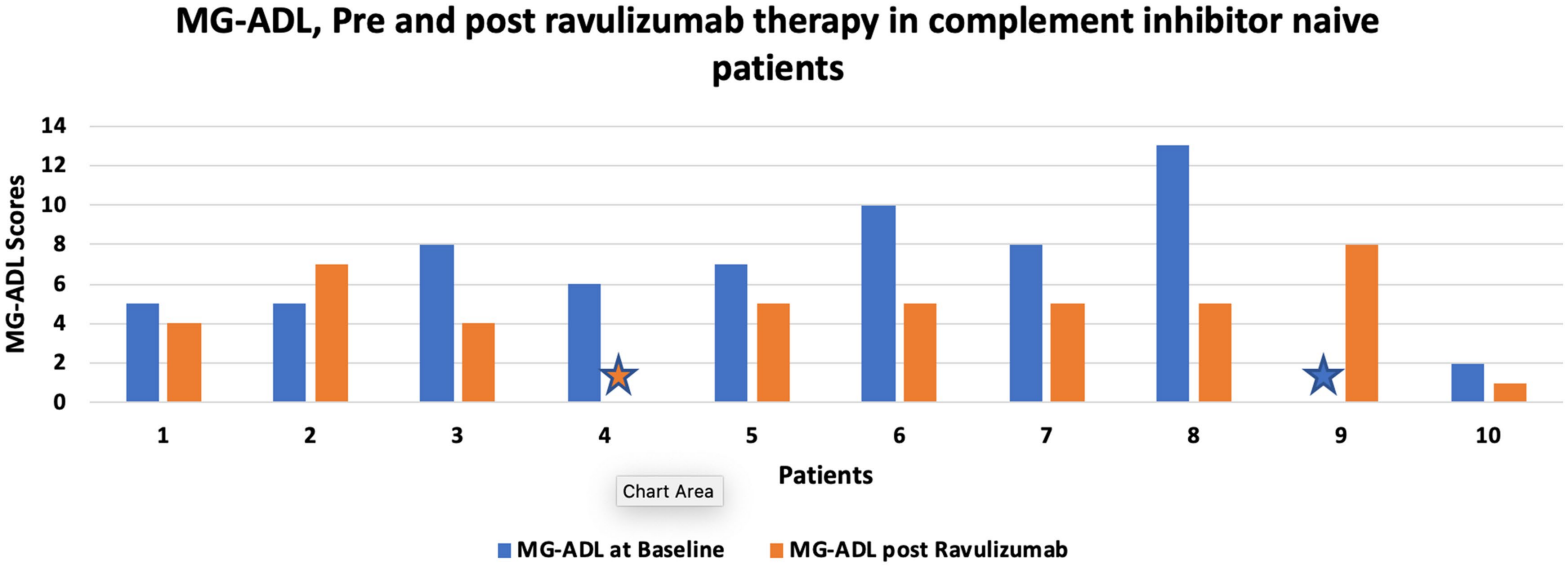
shows change in MG-ADL score, pre and post ravulizumab therapy for complement inhibitor naive patients. Star sign represents MG-ADL score of 0.

Three patients who were switched from efgaritigmod to ravulizumab noted a significant reduction in post ravulizumab mean MG-ADL (Baseline: 7.66 (± 3.7) vs. 5.33 (± 1.24) post ravulizumab). Figure 1 shows change in MG-ADL score, pre and post ravulizumab therapy for complement inhibitor naive patients.

###### 3.2.1.1.2 Reduction in prednisone dose

Eight out of 9, complement inhibitor naive patients were able to reduce their prednisone dosage, post ravulizumab therapy. The mean daily prednisone requirement decreased from baseline dose of 20.44 (± 11.69) mg daily to 15.55 (± 17.21) mg daily therapy over a mean duration of 4.7 (+ 1.20) months.

##### 3.2.1.2 Treatment response in patients switched from eculizumab to ravulizumab

A total of 8 patients were switched from eculizumab to ravulizumab. Four patients with baseline MSE status maintained MSE status post ravulizumab. Out of the 4 patients without baseline MSE status, two patients achieved MSE status post ravulizumab therapy whereas the MG-ADL score remained unchanged for the remaining two patients. The mean time from loading dose to post ravulizumab MG-ADL assessment was 5 (±1.06) months.

**Figure 2:**
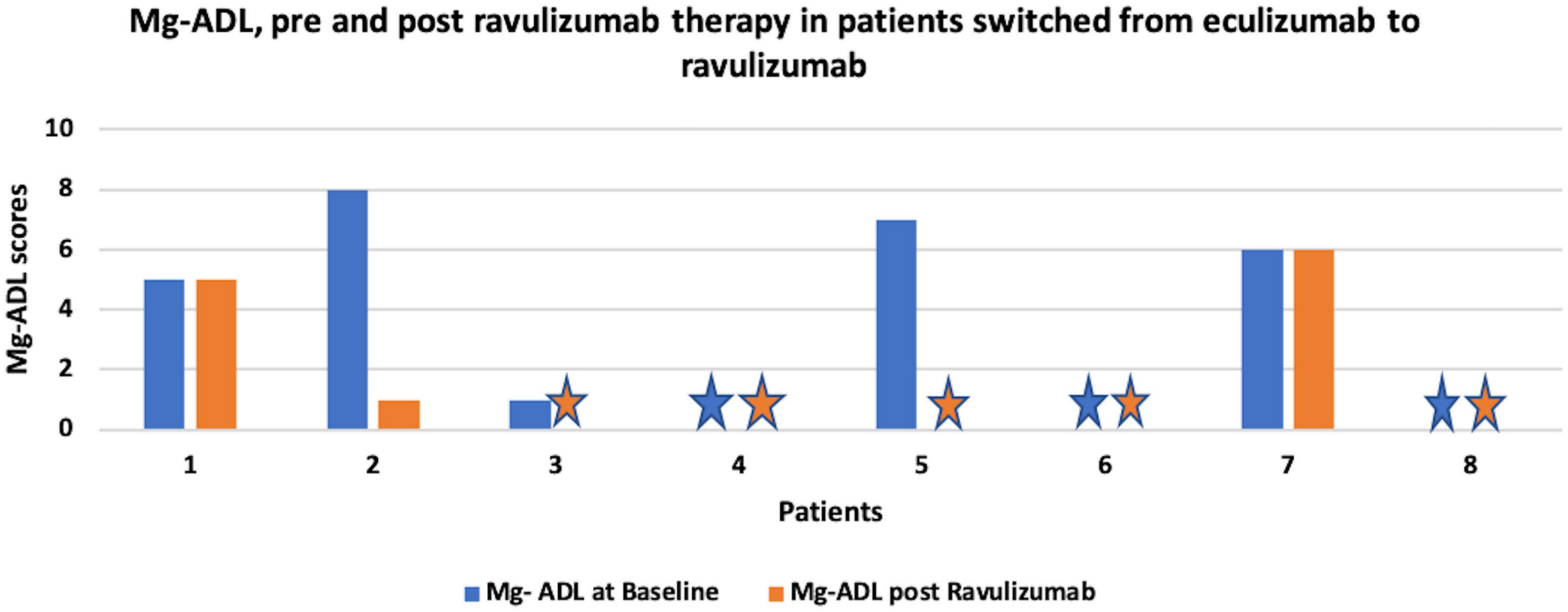
shows change in MG-ADL score, pre and post ravulizumab therapy in patients switched from eculizumab to ravulizumab. Star sign represents MG-ADL score of 0.

Figure 2 shows change in MG-ADL score, pre and post ravulizumab therapy in patients who were switched from eculizumab to ravulizumab.

###### 3.2.1.2.1 Reduction in prednisone dose

Three out of 5 patients switched from eculizumab to ravulizumab were able to reduce their prednisone dosage. The mean daily prednisone requirement decreased from baseline dose of 17 (±24.13) mg/day to 9 (±12.44) mg/day over a mean duration of 4.2 (± 1.09) months.

#### 3.2.2 Other special interest populations

##### 3.2.2.1 Patients previously on IVIG

Two out of three patients were receiving IVIG every 2 weeks prior to initiation of ravulizumab, one patient received IVIG as rescue therapy. One out of 3 patients, who were on IVIG therapy at baseline, noted a clinically meaningful reduction in MG-ADL score. MG-ADL score remained unchanged for one patient and worsened for the other patient.

##### 3.2.2.2 Patients with prior thymoma

One of the two patients with a history of thymoma (s/p thymectomy) achieved MSE, while the other patient maintained MSE status after initiation of ravulizumab.

### 3.3 Side effects

4 patients experienced side effects, 3 had headaches and 1 had an upper respiratory tract infection and diarrhea. All side effects were mild, and patients were able to tolerate further infusions. None of the patients experienced any major side effects. The remaining 14 patients tolerated the infusions well without any documented side effects.

### 3.4 Discontinuation

Ravulizumab was discontinued for one, previously complement naive patient due to worsening MG-ADL. The time duration from loading dose of ravulizumab to discontinuation was 3 months.

## 4 Discussion

This retrospective case series highlights our experience with ravulizumab in AChR+ve generalized MG patients. In our cohort, more than 60% of complement inhibitor naïve patients had a clinically meaningful reduction in MG-ADL. Additionally, patients who were switched from eculizumab had a further reduction in MG-ADL. Finally, 78% of patients were able to reduce their prednisone dose after starting ravulizumab, revealing a steroid-sparing effect within a few months.

With dramatic interest in the development of novel therapies for MG, we expect increasing options in complement inhibition in patients with AChR+ve MG. Currently, two intravenous monoclonal antibody therapies, eculizumab, and ravulizumab with different half-lives are FDA approved. Another therapy, Zilucoplan, a peptide that binds to C5 and prevents the formation of a membrane attack complex, given daily by the subcutaneous route, received FDA approval in October 2023 (1). Additional oral and IV complement inhibitor therapies are in development (9).

With all these expensive therapeutic options, it is critical to identify the patients most likely to benefit from complement inhibition. Multiple case–control studies have evaluated markers of complement activation including CH50, byproducts of complement activation, but these markers do not correlate with the disease state or clinical responsiveness to complement inhibition. Cell-based analysis of antibodies from patients correlating AChR antibody titers and complement binding has revealed that not all patients with AChR+ve MG have significant complement binding seen only in 46.5% (72/155) serum samples revealing no complement activation (10). Additionally, complement binding did not always correlate with disease severity. More recent analysis suggests that the synergistic action of multiple AChR antibodies targeting different epitomes is critical for complement activation and disease severity (11). Nevertheless, these cell-based complement activation assays are not available for clinicians, therefore, no clear guidance is available for optimal patient selection for complement inhibitor therapy.

Our analysis included patients who were complement inhibitor therapy naïve and those who were already on eculizumab. 60% of complement inhibitor naive patients had a clinically meaningful improvement. Whereas none of the patients who were switched from eculizumab to ravulizumab had any worsening in MG-ADL and all patients maintained the clinical benefits. Interestingly, 2 symptomatic patients who switched from eculizumab to ravulizumab achieved MSE. Unlike clinical trials, where patients would qualify for complement therapy with active symptom burden (MG-ADL above 6), in practice, complement therapies are also used to reduce prednisone dose or simply immunosuppressive regimen.

Our analysis includes many patients who would not otherwise meet phase 3 clinical trial criteria such as patients who are on IVIG or those with a recent history of thymoma. In our study population, one of the two patients with a history of thymoma (s/p thymectomy) achieved MSE, while the other patient maintained MSE status after initiation of ravulizumab. This finding suggests that ravulizumab might be an effective option even in patients with thymoma, similar to publications showing the effectiveness of eculizumab in thymoma patients (12). Additionally, in our analysis, 3 patients were on IVIG. One of three patients on IVIG had clinical improvement in MG-ADL (improved from 7 to 0), this patient also had a history of thymoma. Another patient on IVIG had no change in ADL (5, before and after ravulizumab). The remaining one patient noted worsening of MG-ADL (worsened from 0 to 8). At baseline, this patient had MSE status on prednisone 5 mg daily, azathioprine 50 mg daily and IVIG therapy. He was transitioned to ravulizumab therapy due to the need for frequent IVIG infusions. For a brief period after initiation of ravulizumab, he was able to wean off both prednisone and azathioprine. Unfortunately, he had worsening symptoms, 4 months after loading dose, requiring initiation of prednisone 60 mg daily and azathioprine 50 mg daily. He continued ravulizumab therapy and his MG-ADL improved to 2, 6 months after loading dose of ravulizumab. In our analysis, there were no incidences of severe meningococcal or other severe bacterial infections. All patients were appropriately vaccinated before starting ravulizumab therapy. We did not find any other safety concerns.

Our retrospective analysis has a few limitations. We only obtained MG-ADL score as an outcome measure and there was variation in the timing of MG-ADL assessments among providers. The assessments were performed immediately before and whenever possible, post ravulizumab, and the exact date of clinical outcome was not pre-determined and so we cannot comment on the onset of benefit from ravulizumab. Side effects were only collected from notes and not from standardized case report forms and so are prone to recall bias and only major side effects were documented. Additionally, unlike clinical trials, we had patients who were not actively symptomatic, and therefore assessment of clinical benefit cannot be ascertained only from the MG-ADL scores.

## 5 Conclusion

In summary, this analysis provides evidence of the clinical effectiveness of ravulizumab in diverse clinical populations of AChR+ve MG patients. Further long-term analysis of these patients is needed to assess the persistence of the clinical benefit and need for chronic complement inhibition.

## Data availability statement

The original contributions presented in the study are included in the article/supplementary material, further inquiries can be directed to the corresponding authors.

## Ethics statement

The studies involving humans were approved by Stanford Institutional review board committee. The studies were conducted in accordance with the local legislation and institutional requirements. Written informed consent for participation was not required from the participants or the participants' legal guardians/next of kin because of the retrospective nature of the study.

## Author contributions

NK: Writing – review & editing, Writing – original draft, Visualization, Validation, Project administration, Methodology, Investigation, Formal analysis, Data curation, Conceptualization. RG: Writing – review & editing, Visualization, Validation, Supervision, Methodology, Conceptualization. NG: Writing – review & editing, Validation, Supervision, Conceptualization. SuM: Writing – review & editing, Visualization, Validation, Supervision, Resources, Project administration, Data curation, Conceptualization. SrM: Writing – original draft, Methodology, Investigation, Writing – review & editing, Visualization, Validation, Supervision, Resources, Project administration, Data curation, Conceptualization.
